# Case of Osteo-Sarcoma, Involving the Inferior Portion of the Femur

**Published:** 1839-03-16

**Authors:** John Andrews


*Case of Osteo-Sarcoma, involving the inferior portion of the Femur.* By John Andrews, M. D.

*January* 16*th*, 1838.—I was requested to see, in consultation, Mrs. Rex, residing in the country, about nine miles from town. I found the patient a woman in her fortieth year, the mother of six children, the youngest of which was in its third year. The patient had enjoyed good health until about a year before, when she began to complain of a deep-seated, aching pain, immediately above the left knee. For two or three months no swelling was perceived, and but little done,—the disease being regarded as rheumatism, and treated with frictions of stimulating liniments. In the course of the ensuing spring, a hard tumour was discovered between the hamstrings of the left thigh. The tumour had a "bony hardness," was not painful to the touch, and showed no disposition to inflame; but as it enlarged, the deep-seated pain became more severe and lancinating. Notwithstanding the many and various "washes," "poultices," &c. &c., that were used, the tumour enlarged, particularly *around* the limb, so as to encircle it in the latter part of June. About the 1st of July, in the act of walking across the room, she felt her limb suddenly to give way under her, accompanied with a distinct sound, and excruciating pain. Sinking upon the floor, she was unable to rise, and was carried to her bed, which she had not since been able to leave. From this time, the enlargement of the limb became more perceptible, accompanied with an increase of lancinating pain, especially on the slightest effort to move the limb, producing loss of sleep, emaciation, and, for the last two months, regular hectic paroxysms. At this time, the tumour involved about one-third of the thigh from its lower extremity, extending over the knee, and was of an irregular ovoid shape, terminating abruptly above, less so below, and being about eight inches in its transverse, and twelve in its longitudinal diameter. The skin was very tightly stretched over the tumour, was very transparent, and much marbled by the subjacent veins, which were numerous and large. The surface was uneven, as if covering a lobulated mass; was slightly warmer than natural, very hard, and not sensitive. From the history and present condition of the disease, it was pronounced to be osteo-sarcoma of the os femoris; and amputation was the only *possible* means of relieving the patient. This view of the disease, and the remedy, being made known to the friends and the patient, she was the first to decide in favour of the operation. But it does not follow, even when no other means of relief are presented, that the *ultima ratio* of surgeons should *therefore* be resorted to. A more minute inquiry into the general condition of the patient, disclosed the existence of a scirrhous tumour the size of a hen's egg, in one of the mammæ, and another, somewhat less, on the neck, over the right sterno-cleido-mastoid muscle, to which it appeared to be firmly attached. These appearances rendered the conclusion inevitable that the patient was infected with a constitutional carcinomatous taint, and consequently that no well-founded hope of a perfect cure from the operation could be entertained. The general health was, however, not so much impaired as to make the operation, *per se*, unusually dangerous. All these considerations being fully explained, and a day's reflection recommended to the patient and her friends, she was left, and again visited on the 18th,—when I found her with a mind composed and resolute, resting upon that stronger than all other supports, "the Christian's hope," and decided to operate on the following day. Accordingly, on the 19th I amputated the thigh, performing the circular flap operation, which was borne with great fortitude. The amputated limb was then examined, and the section of the bone included about one and a half inches of healthy bone, below which, including the condyles, the bony structure almost entirely disappeared, its place being supplied by a soft, semifluid mass, in which spicula of bone could be discerned. The cartilages, tendons, muscles, and cellular membrane, were completely transformed into a homogeneous mass, of an encephaloid character, having the colour and general appearances of the cortical portion of the cerebrum somewhat hardened. Sections of the diseased mass were smooth and striated. The stump appeared healthy, with the exception of the cancelli of the bone, which had a dark appearance. It healed very kindly,—and in five weeks after the operation, I had the pleasure of seeing her sitting before the fire, with a cheerful countenance, engaged in knitting. She was now placed upon the comp. syrup of sarsaparilla, with a hope of removing the constitutional taint. But very soon afterwards I had the mortification of seeing the tumour on the neck begin to grow in size, and become more vascular. It assumed a tuberculated form, with a central conoid projection, and acquired, if it had not originally, very deep connexions, apparently with the cervical vertebræ. Several other small tumours appeared over the dorsal vertebræ; but that on the neck grew most rapidly, and attained such a size as to cause the patient's death on the 10th of June following, apparently by pressure on the œsophagus and trachea.

